# Multilevel analysis of geographic variation among correlates of child undernutrition in India

**DOI:** 10.1111/mcn.13197

**Published:** 2021-05-07

**Authors:** Anoop Jain, Justin Rodgers, Zhihui Li, Rockli Kim, SV Subramanian

**Affiliations:** ^1^ Global Health & Social Medicine Harvard Medical School Boston Massachusetts USA; ^2^ Harvard Center for Population and Development Studies Cambridge Massachusetts USA; ^3^ Department of Social and Behavioral Sciences Harvard T.H. Chan School of Public Health Cambridge Massachusetts USA; ^4^ Division of Health Policy & Management, College of Health Science Korea University Seoul South Korea; ^5^ Interdisciplinary Program in Precision Public Health, Department of Public Health Sciences Graduate School of Korea University Seoul South Korea

**Keywords:** child nutrition, international child health nutrition, low‐income countries, nutritional interventions, social factors, socio‐economic factors

## Abstract

Prior research has identified a number of risk factors ranging from inadequate household sanitation to maternal characteristics as important determinants of child malnutrition and health in India. What is less known is the extent to which these individual‐level risk factors are geographically distributed. Assessing the geographic distribution, especially at multiple levels, matters as it can inform where, and at what level, interventions should be targeted. The three levels of significance in the Indian context are villages, districts, and states. Thus, the purpose of this paper was to (a) examine what proportion of the variation in 21 risk factors is attributable to villages, districts, and states in India and (b) elucidate the specific states where these risk factors are clustered within India. Using the fourth National Family Health Survey dataset, from 2015 to 2016, we found that the proportion of variation attributable to villages ranged from 14% to 63%, 10% to 29% for districts and 17% to 62% for states. Furthermore, we found that Bihar, Jharkhand, Madhya Pradesh, and Uttar Pradesh were in the highest risk quintile for more than 10 of the risk factors included in our study. This is an indication of geographic clustering of risk factors. The risk factors that are clustered in states such as Bihar, Jharkhand, Madhya Pradesh and Uttar Pradesh underscore the need for policies and interventions that address a broader set of child malnutrition determinants beyond those that are nutrition specific.

**Key messages**

- Households in India experience concurrent asset and social deprivations that are associated with a whole host of deleterious child and adult health outcomes.
- Current initiatives aimed at addressing these risk factors target districts within India. Additionally, interventions aimed at addressing child malnutrition are typically nutrition‐specific and do not consider a broader set of child health determinants.
- We find that a greater proportion of the variation for 19 of the 21 risk factors included in this study is attributable to villages, suggesting that interventions should also target this geographic level.
- Furthermore, based on the risk factors that were clustered in Bihar, Jharkhand, Madhya Pradesh and Uttar Pradesh, our findings underscore the need for interventions to address a broader set of child health determinants beyond those that are just nutrition specific. This is important given that each of these four states is consistently among the worst performing in terms of child health outcomes in India.

## 1 INTRODUCTION

Poverty is a multidimensional construct in which people experience concurrent deprivations across various health, education and standard of living indicators (Initiative et al., 2019). Impoverished households are often food insecure, lack access to essential health services, and are unable to invest in education (Galobardes et al., 2007; Karlsson et al., 2020; Victora et al., 2003). In turn, these deprivations are risk factor for adverse child health outcomes. For example, child malnutrition remains a major issue in India where in 2017, the prevalence of child stunting was 39.9%, child wasting 15.7%, and child underweight 32.7% (Swaminathan et al., 2019), and can be linked to multigenerational poverty and inadequate environmental conditions (Corsi et al., 2016; Kim et al., 2019; Martorell & Young, 2012). Furthermore, easy access to durable goods and amenities can indirectly lead to improved child health outcomes by increasing time for direct childcare, ensuring good household hygiene, and economic activities (Greenwood et al., 2005; Lewis, 2018; Mokyr, 2000). Additionally, poor maternal nutrition before and during pregnancy is associated with child stunting (Coffey, 2015), underscoring the importance of socio‐economic status (SES) and the consumption of basic necessities early in a mother's life as a means of preventing growth faltering in the subsequent generation (Perkins et al., 2016; Subramanian, 2009).

Asset and social deprivations are risk factors for outcomes outside the realm of child health, too. For example, women and girls living in homes without access to potable water and clean cooking fuel are often assigned the responsibility for fetching drinking water and firewood [World Health Organization (WHO) 2016b; WHO, 2017]. Women and girls who carry water and firewood from long distances are at greater risk for infection, injury, physical and sexual violence, and chronic musculoskeletal problems (Geere et al., 2018; WHO, 2017). Additionally, inadequate access to sanitation has been linked to lower well‐being scores, and higher anxiety, depression, and distress thereby underscoring the intrinsic value of household sanitation (Caruso et al., 2018; Jain & Subramanian, 2018). Finally, evidence suggests that investing in women's education is essential to transform gender norms (Heise et al., 2019; Malhotra et al., 2019), which can reduce exposure to other risk factors thus leading to reductions in gender‐based health disparities (Victora et al., 2003).

There is a need to understand the geographic distribution of these risk factors in India from a multilevel perspective given that the unique mechanisms may operate at each level and interact with one another to generate different patterns of clustering. Previous studies that have examined how poverty varies across single geographic levels in India have largely overestimated the importance of that particular level. For example, studies have examined poverty variation across states in India (Alkire & Seth, 2015; Baddeley et al., 2006; Dev & Ravi, 2007). Other studies have looked at the variation in deprivations across districts (Banerjee et al., 2015; Chaudhuri & Gupta, 2009). However, a more recent study employing multilevel modelling points to the importance of village level factors, in addition to state‐level context, in shaping the distribution of household deprivations across India (Kim et al., 2016). Thus, multilevel studies are important given that studying variation at only one level may lead to an overestimation of that particular level's effect.

Given this background, the purpose of this paper was to conduct a multilevel analysis using data from India's most recent National Family Health Survey (NFHS) survey from 2015 to 2016 to: (a) examine what proportion of the variation in 21 risk factors is attributable to villages, districts, and states in India and (b) elucidate which states these risk factors are most highly clustered in. The risk factors selected for this study, to our knowledge, are the most comprehensive list of risk factors that are known to be associated with child malnutrition (Kim et al., 2019; Menon et al., 2018). These risk factors were selected based on the fact that they have been found to be associated with negative child health outcomes according to the work done in prior studies ( Corsi et al., 2016; Kim et al., 2019; Ruel & Alderman, 2013).

This research is significant for several reasons. First, geographic clustering of poverty and other disadvantages is important in understanding area effects on health (Diez Roux, 2001; Gephart, 1997). This is particularly salient in the Indian context given regional variations in health outcomes such as child malnutrition (Cavatorta et al., 2015; Menon et al., 2018; Rodgers et al., 2019). Therefore, it is important to understand specific regions within India, where child malnutrition risk factors are clustered. Doing so will inform the specific states that policies should target in order to improve child malnutrition outcomes. Furthermore, the National Institution for Transforming India (NITI) Aayog's National Nutrition Strategy (NNS) focuses on district‐level interventions to improve child nutrition outcomes [National Institution for Transforming India (NITI) Aayog, 2017]. Elucidating which geographic level the variation in each of these 21 risk factors is attributable to will help better inform which geographic level policies should be targeted at, and the specific risk factors that should be targeted at that level.

## 2 METHODS

### 2.1 Data source

We used the NFHS data set, which is from 2015 to 2016. Overall, this data set contains data from each of India's 36 states/union territories, all 640 districts, 28,522 sampled clusters out of over 650,000 villages, 699,686 women between the ages of 15–49 (primary respondents), and 259,627 children between the ages of 0–59 months. The survey used a stratified two‐stage sampling frame (by states, and urban and rural areas within states) to select participants [International Institute for Population Sciences (IIPS), 2018]. The aim of the NFHS was to achieve a representative sample of 15% of households in India, which was a done by surveying 22 households per PSU (IIPS, 2018). A detailed outline of the sampling strategy is described in the NFHS documentation (IIPS, 2018). We restricted our sample to one child per household. As such, we included 180,209 children and households in our sample from all 36 states/union territories, 640 districts, and 28,332 villages.

### 2.2 Outcomes

We included 21 different risk factors to be analysed in this study. We dichotomized using cut‐offs defined in previous studies (Kim et al., 2019) for each of the 21 variables. The definition for each of these variables is further described in Table S1.

#### 2.2.1 Nutrition risk factors

We included four different nutrition variables, child dietary diversity, early breastfeeding initiation, vitamin A supplementation, and the use of iodized salt. Dietary diversity, which was only for children between the ages of 6–23 months, was dichotomized above and below four food groups eaten in the past 24 h. Early breastfeeding initiation (within 1 h of birth), vitamin A supplementation, which was only asked in reference to children between the ages of 6 and 59 months, and use of iodized salt were all dichotomized as yes/no.

#### 2.2.2 Environmental risk factors

We included four environmental variables, household access to improved sanitation, household access to improved drinking water, household air quality, and stool disposal. Household sanitation and drinking water source were dichotomized based on whether the infrastructure was unimproved or improved. For sanitation, unshared facilities that are flushed to a sewer system, septic tank, or pit latrine, and composting toilets, pit latrines with slabs, or ventilated improved pit latrines are all considered improved. For drinking water sources, piped water sources either to the home or public, protected wells, deep borewells or rainwater are all considered improved. Indoor air quality was dichotomized based on fuel type used for cooking, either solid fuel (poor air quality) or non‐solid fuel (good air quality). Finally, we dichotomized stool disposal as safe or unsafe.

#### 2.2.3 Health coverage risk factors

We examined seven health coverage risk factors. These were whether the child experienced an infectious disease—such as diarrhoea or cough/cold—in the past 2 weeks, provision of oral rehydration therapy (ORT) to a child with diarrhoea in the past 2 weeks, whether childbirth was attended by a skilled attendant, whether family planning needs were met for the mother, whether care was sought for a child with symptoms of pneumonia in the past 2 weeks, number of antenatal care (ANC) visits, and child vaccinations. Infectious disease in the past 2 weeks, provision of ORT, presence of skilled attendant at birth, whether or not family planning needs were met for the mother, and care sought for child pneumonia were all dichotomized as yes/no. Given that this NFHS survey is from 2015 to 2016, we used older World Health Organization (WHO) guidelines that recommended at least four ANC visits and as such dichotomized this variable as above and below four visits (WHO, 2016a). The number of ANC visits was only asked in reference to a mother's last birth. The NFHS survey restricted vaccination questions to children between the ages of 12 and 23 months, and we dichotomized vaccinations yes/no based on whether the child had received all vaccinations for measles, BCG, DPT 3 and Polio 3.

#### 2.2.4 Socio‐economic risk factors

We included three SES variables. These were household wealth, mother's level of education, and child's birth order. We used the wealth index score provided by the Demographic Health Survey, which was then dichotomized by households in quintile 1 (poorest) and households in all other quintiles. Mother's education was dichotomized based on whether the mother had received primary or above education. Birth order was dichotomized as before or after the sixth birth.

#### 2.2.5 Maternal characteristic risk factors

Finally, we included three maternal characteristic variables. These were mother's age at marriage, maternal height and maternal body mass index (BMI). Mother's age at marriage was dichotomized as above and below 18 years old (below being the risk factor). Maternal height and BMI were dichotomized above and below 145 cm, and above and below 18.5 kg/m^2^ respectively (below being the risk factor in both cases).

### 2.3 Geographic exposures

The primary exposures in this study were states/union territories, districts, and villages that any given household is nested in (the number of villages and districts by state is described in Table S2). In doing so, we build off previous work that examined within and between‐population variations in child anthropometric failures in India (Rodgers et al., 2019). Each of these three geographic levels is uniquely important in shaping the distribution of child malnutrition risk factors. Federal policies by India's central government operate at the state/union territory level, whereas districts are the lowest administrative unit where planning for the provision of various services occurs (Kim et al., 2016). Villages are the most local social, political, and economic environments. Therefore, they carry far more significance than simply being primary sampling units (IIPS, 2018).

### 2.4 Statistical analysis

Our data structure was such that children at Level 1 were nested in villages at Level 2, districts at Level 3, and states at Level 4. In order to decompose the geographic variation attributable to Levels 2, 3, and 4, we estimated 21 different four‐level variance component models (VCMs) for the probability (*Pr*) of a child *i* in village *j*, district *k,* and state *l* exposed to the given risk factor as shown in Equation 1

(1) $$\text{logit}\left({\mathrm{Pr}}_{\text{ijkl}}\right)=\beta_{0}+\left(u_{0\mathrm{jkl}}+v_{0\mathrm{kl}}+f_{0l}\right).$$

In this model, we estimated the log odds of the risk factor, where *β*_0_ represents an average child in a village, district, and state with zero random effects. The random effects are interpreted as residual differentials for villages *j* (*u*_0*jkl*_), districts *k* (*v*_0*kl*_), and states *l* (*f*_0*l*_). Each residual differential is assumed to be normally distributed with a mean of 0 and a variance of *u*_0*jkl*_ ~ $N(0,\sigma_{u0}^{2}$), *v*_0*kl*_ ~ $\;N(0,\sigma_{v0}^{2}$), and *f*_0*l*_ ~ $\;N(0,\sigma_{f0}^{2}$). Variance component models are used to measure the proportion of variance attributable to the state, district, and village levels (Goldstein, 2011). The variances quantify the between‐village ( $\sigma_{u0}^{2})$ between‐district ( $\sigma_{v0}^{2}),$ and between‐state ( $\sigma_{f0}^{2})$ variations in the log odds of child *i* experiencing a given risk factor. The variance of the lowest level (children in this case) cannot be calculated in models with binary outcomes, and the remaining variance is a function of the binomial distribution (Kim et al., 2016). We summed each of these three variances to calculate a total value for the geographic variation for each risk factor. We then partitioned the variance at each of the three geographic levels by dividing the variance of a given level by the total geographic variation (i.e., for village level, $\sigma_{u0}^{2}$/( $\sigma_{u0}^{2}$ + $\;\sigma_{v0}^{2}$ + $\sigma_{f0}^{2}$) × 100). This yielded the variance‐partitioning coefficient (VPC), which in this case represents the percentage of variation for a given risk factor attributable to one of the three geographic levels included in our analysis. The VPC is one of the primary parameters of interest when analysing VCMs and is interpreted as the magnitude of variability across different units of inference (geographic levels, in this case) (Kawachi & Berkman, 2003).

We then repeated this process for 21 different three‐level models in each state such that children at Level 1 were nested in villages at Level 2, and districts at Level 3. In this case, we decomposed the variation attributable to Levels 2 and 3 for the probability (*Pr*) of a child *i* in village *j* and district *k* exposed to the given risk factor as shown in Equation 2:

(2) $$\text{logit}\left({\mathrm{Pr}}_{\mathrm{ijk}}\right)=\beta_{0}+\left(u_{0\mathrm{jkl}}+v_{0\mathrm{kl}}\right).$$

The same assumptions and parameter definitions as noted above were applied to this model. We then partitioned the variance at the village and district levels by dividing the variance of a given level by the total geographic variation (i.e., for village level, $\sigma_{u0}^{2}$/( $\sigma_{u0}^{2}$ + $\sigma_{v0}^{2}$) × 100). Again, this yielded the VPC, which in this case represents the percentage of variation for a given risk factor attributable to either the village or the district levels in each state. We excluded Chandigarh, Dadra and Nagar Haveli, and Lakshadweep from the state‐specific analysis as these Union Territories only have one district.

Finally, we used the posterior state‐level residuals calculated from Equation 1 *f*_0*l*_ for each risk factor to create quintile bins from the lowest 20% to the highest 20% of the residual distribution. In multilevel modelling, residuals can be interpreted as individual estimates, or the random intercepts, for any given level (in this case, states) (Goldstein, 2011). The bins described which states were at highest, high, moderate, low, and lowest risk for each child health risk factor. Organizing the results in this way allowed us to elucidate the extent to which the risk factors are clustered in any given state. We used MLwiN 3.05 (University of Bristol, Bristol, United Kingdom) to conduct the VCM analysis and estimate the residuals using iterative generalized least squares.

## 3 RESULTS

### 3.1 Sample characteristics

We analysed data from 180,209 children and households. These households were nested in 28,332 villages, 640 districts, and 36 states/union territories. Of the 52,735 children between the ages of 6 and 23 months, 44,497 (84%) ate less than four food groups in the past 24 h, and 92,889 (55%) of all children were not breastfed within 1 h of their birth. Additionally, 69,168 (44%) children between the ages of 6 and 59 months were not receiving vitamin A supplementation, and 10,254 (6%) lived in households not using iodized salt. In terms of household sanitation, 86,866 (48%) households did not have access to improved sanitation, and 21,156 (12%) households used an unimproved water source. In terms of cooking fuel, 121,485 (67%) households used solid fuel sources, and 113,609 (65%) households did not safely dispose of children's stool. In our sample, 40,477 (23%) children had an infectious disease in the past 2 weeks. Of the 16,373 children who had diarrhoea in the past 2 weeks, 6583 (40%) did not receive ORT, and 37,774 (21%) children were born in the absence of a skilled birth attendant. Among the mothers in our sample, 49,866 (28%) reported that their family planning needs were unmet. Additionally, 2516 (25%) of the 9960 children who had symptoms of pneumonia in the past 2 weeks did not receive any treatment. Among the mothers in our sample, 76,200 (51%) went to fewer than four ANC visits. Of the 35,217 children between the ages of 12 and 23 months, 13,210 (38%) were not fully vaccinated. Of the 180,209 households in our sample, 45,195 (25%) were in the poorest wealth quintile, and 52,738 (29%) mothers reported not receiving any education. Additionally, 7402 (4%) children in our sample were the sixth or more birth in the family, and 62,882 (36%) mothers were married before their 18th birthday. In terms of maternal height and BMI, 19,906 (11%) mothers were less than 145 cm, and 40,596 (23%) mothers had a BMI less than 18.5. Table 1 below presents the full geographic hierarchy and distribution of risk factors included in our analysis.

**TABLE 1:** Geographic hierarchy and distribution of risk factors included in analysis

Category	Risk factor	States	Districts	Villages	Eligibility criteria	Number of eligible children	Missing children	Total children	Risk factor	Number of at risk children	Percent of at risk children (%)
Nutrition‐specific risk factors	Delayed breastfeeding	36	640	28,265		180,209	10,370	169,839	>1 h	92,889	55
	Non‐iodized salt	36	640	28,327		180,209	535	179,674	Not used	10,254	6
	No vitamin A supplementation	36	640	28,224	6–59 months	157,800	0	157,800	No	69,168	44
	Poor dietary diversity	36	640	23,394	6–23 months	52,735	0	52,735	<4 food group	44,497	84
Environmental risk factors	Unsafe drinking water	36	640	28,332		180,209	0	180,209	Unimproved	21,156	12
	Solid cooking fuel	36	640	28,332		180,209	17	180,192	Solid fuel	121,485	67
	Unimproved sanitation	36	640	28,332		180,209	0	180,209	Unimproved	86,866	48
	Unsafe stool disposal	36	640	28,304		180,209	5920	174,289	Unsafe	113,609	65
Health coverage risk factors	No care after pneumonia	36	632	7064	Pneumonia in past 2 weeks	9960	0	9960	No care	2516	25
	No safe birth attendant	36	640	28,329		180,209	86	180,123	Not present	37,774	21
	No ORT after Diarrhoea	36	638	10,488	Diarrhoea in past 2 weeks	16,373	0	16,373	Not used	6583	40
	Unmet family planning needs	36	640	28,322		180,209	1802	178,407	Not met	49,866	28
	Not fully vaccinated	36	640	19,758	12–23 months	35,217	0	35,217	No	13,210	38
	Less than 4 ANC visits	36	640	28,193		180,209	30,534	149,675	<4 visits	76,200	51
	Infectious disease in past 2 weeks	36	640	28,320		180,209	3802	176,407	Yes	40,477	23
SES risk factors	Poorest quintile (wealth)	36	640	28,332		180,209	0	180,209	Poorest quintile	45,195	25
	Birth order	36	640	28,332		180,209	0	180,209	Sixth or more	7402	4
	Mother not educated	36	640	28,332		180,209	0	180,209	No schooling	52,738	29
Maternal risk factors	Short stature mother	36	640	28,305		180,209	2214	177,995	<145 cm	19,906	11
	Low BMI mother	36	640	28,304		180,209	2442	177,767	<18.5	40,596	23
	Mother married <18	36	640	28,315		180,209	3140	177,069	<18	62,882	36

### 3.2 Variance partitioning across India

**FIGURE 1:**
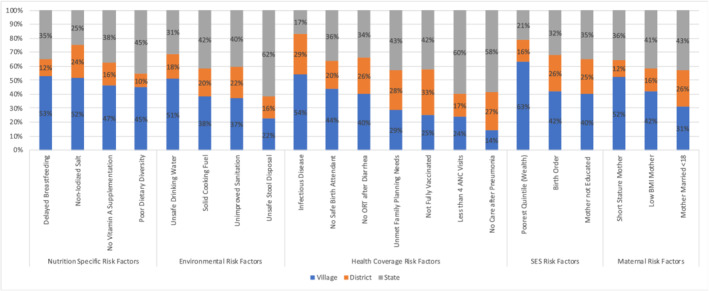
Partitioning of geographic variance for 21 child health risk factors by village, district and state

We analysed 21 different four‐level VCMs, one for each risk factor. We used these results to partition the variance by geographic level to demonstrate the percentage of variation in a risk factor that is attributable to states, districts and villages (Figure 1). Overall, we found that the greatest proportion of variation was attributable to villages, then states, then district for 12 of the 21 risk factors. For one of the 21 risk factors (infectious disease), the greatest proportion of variation was attributable to villages, then districts, then states. In six of the 21 risk factors, the greatest proportion of variation was attributable to states, then villages, then districts. We found that the proportion of variation attributable to villages ranged from 14% (no care sought for pneumonia) to 63% (households in the poorest wealth quintile). The proportion of variation attributable to districts ranged from 10% (dietary diversity) to 29% (infectious disease), and the proportion of variation attributable to states ranged from 17% (infectious disease) to 62% (unsafe stool disposal).

### 3.3 Variance partitioning across villages and districts by state

**FIGURE 2:**
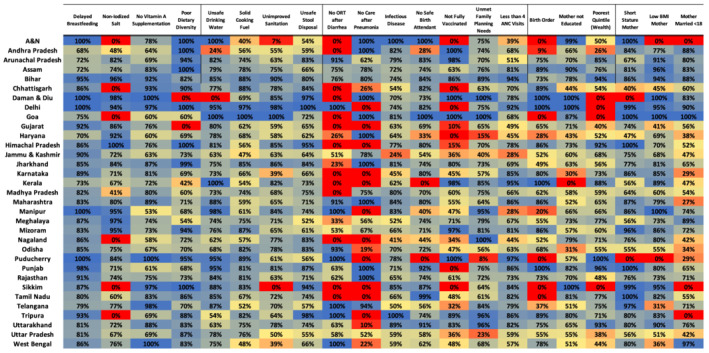
Proportion of variation attributable to villages by states across India for each risk factor. Proportion of variation attributable to districts is 100 minus the village value presented in this figure

We analysed 21 different three‐level VCMs to estimate the VPC across villages and districts by state throughout India. These results are presented in Figure 2. The highest VPC value observed for villages was 100% for each of the 21 risk factors. The lowest VPC values observed at the village level ranged from 0% to 8% in 16 of the 21 risk factors (delayed breastfeeding, non‐iodized salt, poor dietary diversity, unsafe drinking water, unimproved sanitation, infectious disease in the past 2 weeks, incomplete vaccinations, no safe birth attendant, no ORT after diarrhoea, no care sought for pneumonia, no safe birth attendant, child not fully vaccinated, birth order, mother's education, household in the poorest wealth quintile, short maternal stature, low maternal BMI and mother's age of marriage). The lowest VPC values at the village level ranged from 24% to 53% in the remaining five risk factors (no vitamin A supplementation, solid cooking fuel use, unsafe stool disposal, infectious disease in past 2 weeks and less than four ANC visits).

The lowest VPC value observed for districts was 0% for each of the 21 risk factors at the district level except for women who went to less than four ANC visits (3% for Puducherry). The highest VPC values at the district level ranged from 32% to 93% in 12 of the 21 risk factors (delayed breastfeeding, vitamin A supplementation, poor dietary diversity, unsafe drinking water, solid cooking fuel use, unimproved sanitation, unsafe stool disposal, infectious disease, no safe birth attendant, unmet family planning needs, less than four ANC visits, and mother's education). The highest VPC value observed at the district level was 100% in nine of the 21 risk factors (non‐iodized salt, no ORT after diarrhoea, no care after pneumonia, not fully vaccinated, birth order, household in the poorest wealth quintile, short maternal stature, low maternal BMI, and mother's age of marriage).

### 3.4 State‐level risk factors

We calculated the residuals for each state for each of the 21 risk factors in our analysis before creating quintile bins for these values to elucidate the states that were in the highest, high, moderate, low, and lowest risk quintiles for each risk factor.

**FIGURE 3:**
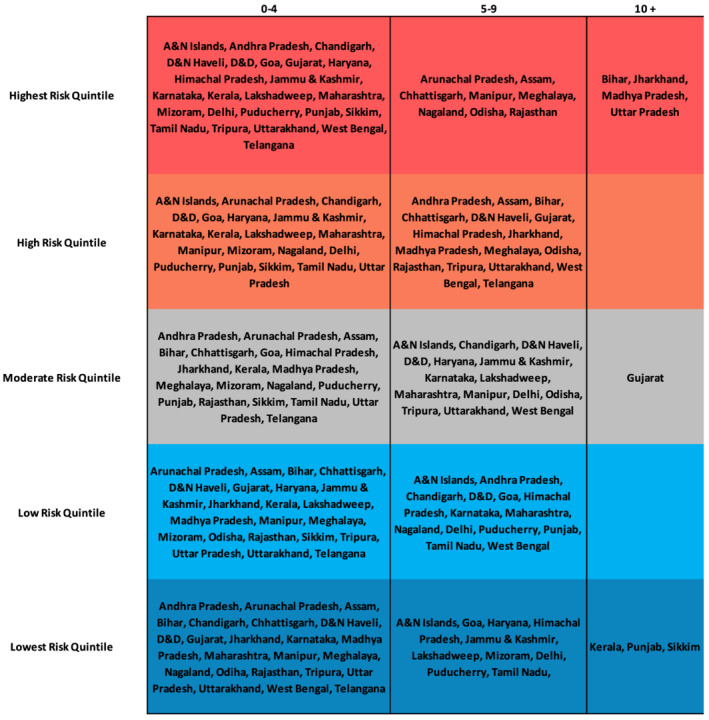
States that were in the lowest, low, moderate, high and highest risk quintiles for 0–4, 5–9 and 10 or more risk factors

We found that Bihar, Jharkhand, Madhya Pradesh, and Uttar Pradesh were in the highest risk quintile for more than 10 of the 21 risk factors as shown in Figure 3. Bihar was among the highest risk quintile states in 12 risk factors, whereas Madhya Pradesh, Jharkhand, and Uttar Pradesh were among the highest risk quintile states in 13, 13, and 15 risk factors, respectively.

**FIGURE 4:**
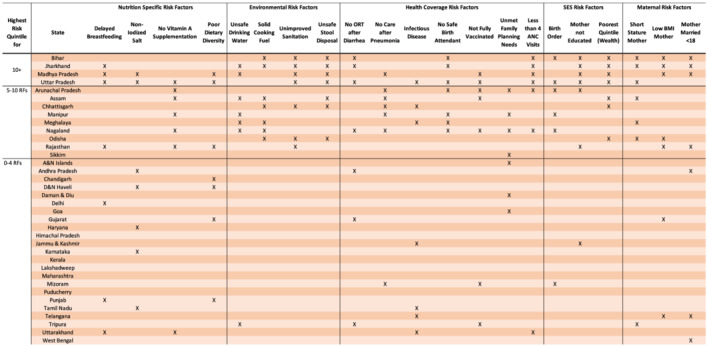
Highest risk quintiles states in (a) 0–4 risk factors, (b) 5–10 risk factors and (c) more than 10 risk factors

The most prevalent risk factor categories were environmental, SES, and maternal. All four states were in the highest risk quintile for unimproved sanitation, unsafe stool disposal, less than four ANC visits, mother's education, and household in the poorest wealth quintile. Bihar, Jharkhand, and Madhya Pradesh were in the highest risk quintile for low maternal BMI and mother's age of marriage. A full list of risk factors clustered by state is noted in Figure 4.

## 4 DISCUSSION

This study had five salient findings. Overall, the greatest proportion of variation in the four‐level models was attributable to villages in 13 of the 21 risk factors. The greatest proportion of variation was attributable to states for the remaining eight risk factors in the four‐level models. In the state‐specific analysis, the highest VPC for villages was 100% for all 21 risk factors in the three‐level models. The lowest VPC values ranged from 0% to 8% in 16 of the 21 risk factors, and 24% to 53% in the remaining five risk factors. At the district level, the lowest VPC value was 0% for all 21 risk factors in the three‐level models except for women who went to less than four ANC visits. The highest VPC values among districts ranged from 32% to 93% in 12 of the 21 risk factors, and 100% in the remaining risk factors. We found that Bihar, Jharkhand, Madhya Pradesh, and Uttar Pradesh were in the highest risk quintile in more than 10 of the 21 risk factors, an indication of risk factor clustering.

There are two data limitations in this study. Although child anthropometric data were collected by trained field staff, responses to questions about the risk factors were self‐reported by the mothers, thereby introducing a potential source of measurement error. Despite this concern, data from the NFHS is widely regarded as high quality and representative (Corsi et al., 2012). Additionally, despite removing Union Territories with only one district, we still found that the lowest VPC value at the district level was 0% for each of the risk factors except women who went to less than four ANC visits. This is likely due to the fact that smaller states such as Goa have a smaller number of districts. The same was true at the village level. Thus, the VPC results small states/union territories are biased due to low intra‐state geographic variation.

Our findings have several policy‐relevant implications. For example, NITI Aayog's NNS implements interventions at the district level as a means of addressing child malnutrition [National Institution for Transforming India Aayog (NITI), 2017]. The results from our four‐level models, however, suggest that the greatest proportion of variation is attributable to villages for 13 of the 21 risk factors associated with child malnutrition. The greatest proportion of variation was attributable to states for the remaining eight risk factors. Our state‐specific three‐level models were also consistent with these results as the average variation attributable to villages for each risk factor was higher for villages than districts. When combined, these findings are similar to results from prior studies that indicate the relative importance of villages and states over districts as the geographic units responsible for variation in risk factors (Kim et al., 2016, 2018; Mohanty et al., 2018). Therefore, although we are unable to identify which specific villages should be targeted, our results suggest that at a high level, villages and states should be considered as geographic units of intervention for policies aimed at addressing the risk factors of malnutrition. Different risk factors will require different interventions. Salt iodization efforts might be very different than efforts to reduce poverty, for example. In terms of feasibility of intervening at the village level, the Integrated Child Development Services (ICDS) programme in India allows for the delivery of essential health and social services in villages throughout India. The ICDS programme utilizes community health workers, known as Anganwadi Workers (AWWs), to deliver nutrition education and supplementation, vaccinations, family planning counselling, basic medicines and a variety of other services at the village level. There are nearly 1.4 million Anganwadi centres in villages across India, each of which serves between 800 and 1000 children along with lactating mothers in its respective catchment area (Rao & Kaul, 2018). Thus, the Anganwadi system implemented by ICDS along with Village Health Sanitation and Nutrition Committees, implemented by the National Health Mission (Ved et al., 2018), underscore the feasibility of delivering vital health services to millions of women and children at even the lowest geographical levels within India. This would complement efforts at the state and district levels being implemented by NNS.

Despite the fact that overall our results point to the relative importance of villages and states over districts with regards to which geographic unit should be targeted, the granularity of our results points to specific states that do not follow this trend for certain risk factors. In Haryana, Jharkhand, and Meghalaya, a greater proportion of the variation for ORT after diarrhoea was attributable to districts than villages. Similarly, a greater proportion of variation for incomplete vaccinations was attributable to districts than villages in states such as Arunachal Pradesh, Gujarat, Jammu and Kashmir, Manipur, Telangana and others. In West Bengal, a greater proportion of variation was attributable to districts than villages for unimproved sanitation. This is consistent with findings from previous studies that have found significant inter‐district socio‐economic disparities, particularly in north and central India (Ohlan, 2013). Thus, our findings suggest that the decision about which geographic unit health interventions should be targeted at depends on the state and the risk factor.

Finally, findings from previous studies show that Bihar, Jharkhand, Madhya Pradesh, and Uttar Pradesh are among the worst performing Indian states in terms of child malnutrition outcomes (Hemalatha et al., 2020; Swaminathan et al., 2019). Our findings show that each of these were the only states in the highest risk quintile for more than 10 of the 21 risk factors for child undernutrition. Five of the risk factors were common to all four states. These were unimproved sanitation, unsafe stool disposal, less than four ANC visits, mother's education, and household in the poorest wealth quintile. It is important to note that none of these are nutrition‐specific risk factors. Only Uttar Pradesh was in the highest risk quintile for no vitamin A supplementation. Both Uttar Pradesh and Madhya Pradesh were in the highest risk quintile for households not using iodized salt. Both of these states were in the highest risk quintile for low child dietary diversity. Jharkhand, Madhya Pradesh, and Uttar Pradesh were all in the highest risk quintile for delayed breastfeeding initiation, which has several possible explanations. For example, 37% of women reported experiencing spousal violence in 2017 (IIPS, 2017), a known risk factor for delayed breastfeeding initiation (Young et al., 2019). Furthermore, breastfeeding counselling during pregnancy is associated with early breastfeeding initiation (Young et al., 2019). Yet we show that women were less likely to go to at least four ANC visits all four of these states. Therefore, these findings further underscore the need for policies and interventions that address a broader set of child malnutrition determinants beyond those that are nutrition specific (Corsi et al., 2016; Martorell & Young, 2012).

## 5 CONCLUSION

The purpose of this study was to examine what proportion of the variation in 21 nutrition‐specific and nutrition‐sensitive child malnutrition risk factors is attributable to villages, districts, and states in India, and to examine the states where these risk factors are highly clustered within India. Overall, we found that the greatest proportion of variation for risk factors we included was attributable to villages. However, in some states, and for certain risk factors, a greater proportion of the variation was attributable to districts. Thus, our findings suggest that the decision about which geographic unit health interventions should be targeted at depends on the state and the risk factor. We also showed that Bihar, Jharkhand, Madhya Pradesh and Uttar Pradesh are all in the highest risk quintiles for more than 10 risk factors, an indication of risk factor clustering. This is consistent with other findings that show these four states are among the worst performing in terms of child malnutrition. Moreover, our results show that these states were in the highest risk quintiles for environmental, socio‐economic, maternal, and health coverage risk factors. These results underscore the need for interventions to address a broader set of child malnutrition determinants beyond just nutrition‐specific factors.

## CONFLICTS OF INTEREST

The authors declare that they have no conflicts of interest.

## CONTRIBUTIONS

Conceptualization and design: SVS, AJ, JR and RK. Data acquisition and analysis: AJ, ZL. Data interpretation: AJ, SVS, JR, RK and ZL. Drafting of the manuscript: AJ. Critical revisions to manuscript: SVS, JR, RK and ZL. Overall supervision: SVS.

## Supporting information


**Table S1:** List of risk factors included in the primary and secondary analysis
**Table S2:** Hierarchy of districts, villages, and children by state

## Data Availability

The data that support the findings of this study are openly available in India: Standard DHS, 2015–2016 Dataset at https://dhsprogram.com/data/dataset/India_Standard‐DHS_2015.cfm?flag=0.
