# Supplementary material for: Multilevel analysis of geographic variation among correlates of child undernutrition in India
Source: Matern Child Nutr. 2021 May 7;17(3):e13197. doi: 10.1111/mcn.13197 (PMC8189194; doi:10.1111/mcn.13197)
Supplement: Supplementary file 1 — Table S1: List of risk factors included in the primary and secondary analysis Table S2: Hierarchy of districts, villages, and children by state [file MCN-17-e13197-s001.docx]

| - Timing of breastfeeding initiation | A dichotomous variable for initiating breastfeeding ≥1 hour of birth or <1 hour of birth. Dichotomized as yes/no. |
| --- | --- |
| - Use of iodized salt | A dichotomous variable indicating whether the household used iodized salt. Dichotomized as yes/no. |
| - Vitamin A supplementation | A dichotomous variable indicating whether vitamin A supplementation was given to the child. Dichotomized as yes/no. |
| - Dietary diversity | Based on a 24-hour recall of food intake in the NFHS questionnaire, a score for child’s dietary diversity was developed by assigning 1 point for consumption of milk, meat, lentils, starchy staples, vitamin A fruits, other fruits, dairy, and oils/fats/butter, and the score was grouped into quintiles. Dichotomized above and below four food groups. |
| - Source of drinking water | A dichotomous variable indicating safe source of drinking water for water piped into dwelling or yard/plot, public tap/standpipe, tube well or borehole, protected well or spring, rainwater, and bottled water, and unsafe otherwise. Dichotomized as yes/no. |
| - Household air quality | A categorical variable indicating higher air quality for households using non-solid fuels, lower air quality for using solid fuels in separate kitchen, and the worst quality for using solid fuels in non-separate kitchen. Dichotomized as yes/no. |
| - Sanitation facility | A dichotomous variable indicating improved sanitation facility for households with access to flush to piped sewer system, septic tank, or pit latrine, ventilated improved pit latrine, pit latrine with slab, and composting toilet, and unimproved otherwise. Dichotomized as yes/no. |
| - Stool disposal | A dichotomous variable indicating safe or unsafe disposal of child’s stools. Dichotomized as yes/no. |
| - Oral rehydration therapy (ORT) for diarrhea | A binary variable indicating whether ORT was given for a child with diarrhea. Dichotomized as yes/no. |
| - Care seeking for cough/fever | A binary variable indicating whether care was sought for a child with cough as a proxy measure for care seeking for pneumonia. Dichotomized as yes/no. |
| - Infectious disease | A dichotomous variable indicating whether the child experienced infectious disease (e.g., diarrhea, cough/fever) two weeks prior to the survey. Dichotomized as yes/no. |
| - Skilled birth attendant | Indicator variable was created for births attended by skilled health personnel (doctor, nurse, or midwife). Dichotomized as yes/no. |
| - Full vaccination | A dichotomous variable indicating whether the child was fully vaccinated with measles, BCG, DPT 3, and Polio 3. Dichotomized as yes/no. |
| - Family planning needs | Unmet need for family planning was coded as 1 if woman reported unmet need for spacing or limiting, and 0 otherwise. |
| - Antenatal care (ANC) visits | The number of ANC visits was categorized as <4 or ≥4 based on the new WHO recommendation. |
| - Child’s birth order | Categorized as 1^st^, 2^nd^ or 3^rd^, 4^th^ or 5^th^, and 6^th^ or above. Dichotomized as 6^th^ or above/below. |
| - Maternal education | Categorized in five levels: no schooling, primary, secondary, higher secondary, and college education. Dichotomized no schooling or above. |
| - Household wealth index | In the NFHS-4, household wealth index was created using principal component analyses of household characteristics and assets and categorized into quintiles. Dichotomized above and below poorest wealth quintile. |
| - Maternal height | Women’s height was obtained directly by field interview teams using adjustable Shorr measuring boards, and was categorized as: <145, 145-149.9, 150-154.9, 155-159.9, and 160+ cm. Dichotomized above and below 145 cm. |
| - Maternal BMI | Women’s weight was measured using digital Secascales, and maternal BMI was categorized as <18.5, 18.5-24.9, and 25+ kg/m2. Dichotomized above and below 18.5. |
| - Maternal age at marriage | Defined dichotomously for married or cohabitating mothers using the age of 18 years as cutoff. |

Supplementary Table 1: List of risk factors included in the primary and secondary analysis

Supplementary Table 2: Hierarchy of districts, villages, and children by state
